# Anthropometric Reference Values for the Male Thorax in Mexican Adults: Implications for Chest Surgery

**DOI:** 10.7759/cureus.89137

**Published:** 2025-07-31

**Authors:** Blanca Yadira Arámbula-Sánchez, Erika Barlandas-Quintana, Daniel De Luna-Gallardo, Cuahutemoc Marquez-Espriella, Rodrigo Dávila-Díaz, Abraham Hernán Herrera-Sánchez

**Affiliations:** 1 Plastic and Reconstructive Surgery, Mastoclinic, Mexico City, MEX; 2 Plastic and Reconstructive Surgery, Hospital Central Sur de Alta Especialidad de Pemex, Mexico City, MEX; 3 Plastic and Reconstructive Surgery, ABC Medical Center, Santa Fe Campus, Mexico City, MEX

**Keywords:** anthropometry, chest wall contouring, male chest, mexican population, nipple-areola complex (nac)

## Abstract

Introduction

Anatomical information regarding the nipple-areola complex (NAC) in men is limited. Our objective was to perform thoracic anthropometry in Mexican men and determine whether age and body mass index (BMI) influence the position and size of the NAC.

Materials and methods

Sociodemographic data and direct thoracic measurements were collected from 100 male subjects between January and June 2024 by the same physician. Descriptive statistics, the Mann-Whitney U test, and the Kruskal-Wallis test were applied for independent samples. A p-value < 0.05 was considered statistically significant.

Results

The average age was 29.6 years (±4), and the mean BMI was 25 kg/m² (±2.8). The mean difference between the midpoint of the right arm and the height of the right nipple was 4 mm (±13 mm), and 18 mm (±11 mm) on the left. The ratio between the inter-nipple distance and thoracic width was 60% (±4%), and the ratio between inter-nipple distance and chest circumference was 25% (±1%). No significant differences were found regarding age using the Mann-Whitney U test. However, the Kruskal-Wallis test showed significant differences between BMI groups for NAC size, sternal notch-nipple distance (right and left), inter-nipple distance, thoracic width, thoracic circumference, and the difference between the nipple and arm midpoint bilaterally.

Discussion

Age showed no statistically significant effect on anthropometric parameters. However, the NAC position varied according to BMI; individuals with higher BMI had nipples placed more laterally. Chest width and arm length can be easily measured intraoperatively, allowing real-time adjustments. Ratios such as inter-nipple distance to chest width and nipple-to-arm midpoint distance remained consistent across BMI subgroups, supporting their clinical applicability. These findings suggest that surgeons may use 60% of the chest width as a reliable guide for inter-nipple distance, even in patients with elevated BMI.

Conclusion

By systematically documenting areolar diameter and nipple position in relation to anatomical landmarks, this study contributes valuable quantitative data for plastic surgeons making decisions about optimal NAC placement during gender-affirming surgery (female-to-male) and gynecomastia treatment. These results offer a reproducible, anatomy-based framework for individualized NAC positioning and represent an important step toward establishing region-specific standards in male chest surgery.

## Introduction

While numerous studies provide anthropometric measurements regarding ideal breast shape and nipple positioning in women, there is limited anatomical information specifically addressing the male nipple-areola complex (NAC) [1]. The male chest has distinct anatomical features, including its shape, contour, inframammary fold, and NAC size and position, all of which contribute to visual gender identity [2]. An ideal male chest is not entirely flat but exhibits a defined contour, a natural horizontal fold aligned with the pectoralis major, and a small, prominent nipple with a proportionally masculine areola.

Proper NAC positioning is essential during gender-affirming chest surgery and gynecomastia correction [3]. Surgeons often rely on anatomical landmarks (such as the sternal notch, mid-axillary line, and umbilicus) for nipple placement. Yet variations in body composition may affect optimal positioning.

We hypothesize that NAC positioning and areolar size are influenced by patient height and body mass index (BMI), potentially modifying established proportions. The aim of this study was to evaluate how these anthropometric variables affect NAC configuration and location in the Mexican male population, in order to generate reference values for use in chest masculinization procedures.

Although aesthetic proportions of the female NAC have been widely studied [1-4], far less attention has been paid to the male counterpart, despite increasing demand for accurate male chest reconstruction [5,6]. NAC loss in men is uncommon and often perceived as a minor issue. However, many young male patients, particularly those undergoing bilateral NAC reconstruction in gender-affirming surgery [7-10] or treatment for gynecomastia, seek precise and symmetrical outcomes [6]. Despite this clinical relevance, existing literature on masculinization techniques often lacks detailed planning for NAC design and positioning [11,12].

Currently, no universal guidelines exist for achieving an ideal masculine thoracic appearance. Most decisions regarding NAC placement are made individually by the surgeon and patient [10], and previous efforts to define ideal nipple location have relied on measurements from healthy male volunteers. These formulas are based on anatomical landmarks or body ratios [13]. Yet accurate NAC placement requires meticulous planning and a strong understanding of male thoracic anatomy, as common errors include oversized areolas or nipples placed too high or medially [6].

Early methods relied on intuitive or visual estimation [14], but several authors have proposed more structured approaches. Murphy et al. [15] proposed ratios between the sternal groove, suprasternal notch, pubis, and chest circumference. Mett et al. [16] used Mohrenheim's point and a horizontal line positioned 4-4.5 cm above the inframammary fold to determine nipple level. Atiyeh et al. [17] developed formulas using the golden ratio (0.618) and distances from the umbilicus to the suprasternal notch and anterior axillary fold. Shulman et al. [18] based NAC positioning on patient height, chest circumference, and specific nipple-to-landmark ratios. Beer et al. [6] incorporated chest circumference and sternum length into their method. McGregor's approach involved reference points along the sternal notch, midclavicular line, and the midpoint between the elbow and axillary folds [19,20]. Monstrey et al. [11] emphasized the limitations of absolute measurements, while Tanini et al. [13] noted a consistent anatomical relationship between the NAC and the pectoralis major muscle: on average, 3 cm medial to its lateral border and 2.5 cm above its lower insertion. However, these methods were derived primarily from Caucasian, Middle Eastern, or Asian populations, with no data available for Latin American men.

Moreover, the impact of BMI on NAC positioning remains a topic of debate. While some authors argue that increased BMI may lead to overly superior nipple placement when fixed formulas are used [21], others have reported a tendency for nipples to be positioned more laterally in individuals with higher BMI [5].

To address these gaps, we conducted a cross-sectional study to analyze NAC size and anatomical location in Mexican adult males. Our goal was to produce statistically meaningful, population-specific reference values that could guide individualized intraoperative planning in reconstructive and gender-affirming thoracic surgery.

## Materials and methods

Study design and setting

This was a prospective, observational, cross-sectional study conducted between January and June 2024 at a single center in Mexico City: the outpatient clinic of Hospital Central Sur de Alta Especialidad PEMEX. The protocol was approved by the institutional review board (IRB No. 71/2024) and adhered to the Declaration of Helsinki and local regulations.

Participants and sampling

A non-probabilistic quota sampling strategy was used. All cisgender male patients aged 18-60 years who attended the outpatient service for routine care during the study period were consecutively screened. After eligibility assessment by the principal investigator, each candidate received both verbal and written information; those willing to participate signed a written informed consent form on-site. Five percent of the invited patients declined participation, resulting in a final sample of 100 healthy men. The inclusion criteria were (1) cisgender male; (2) age 18-60 years; and (3) absence of breast pathology. The exclusion criteria were prior breast surgery, gynecomastia, endocrine disorders, transgender status, or severe thoracic trauma.

Sample size calculation

Sample size was estimated using WinEpi (University of Zaragoza, Spain) to detect a difference between groups in the primary endpoint (the inter-nipple-to-chest width ratio), assuming a pilot SD = 0.074, 95% confidence level, 80% power, and an absolute precision of 0.020. The calculation required 97 subjects; 100 were recruited to compensate for potential attrition.

Anthropometric measurements

**Figure 1:**
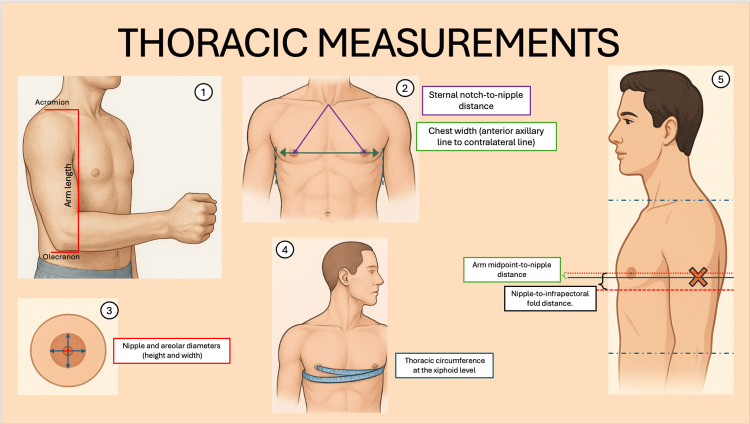
Anthropometric parameters used to assess the nipple-areola complex (NAC) position and proportions in the male thorax. Diagram illustrating the thoracic and upper limb measurements collected in the study. (1) Arm length: measured from the acromion to the olecranon; (2) Chest width (anterior axillary line to contralateral line) and sternal notch-to-nipple distances; (3) Areolar and nipple diameters (height and width); (4) Thoracic circumference measured at the xiphoid level; (5) Vertical relationships between the arm midpoint and nipple, and between the nipple and the infrapectoral fold. These anatomical landmarks support the proportional analysis and intraoperative guidance of NAC placement in male chest surgery. Original figure created by the authors.

All assessments were performed once, during the morning shift (08:00-13:00 h) in a private examination room. Participants stood upright without a shirt. Equipment included a calibrated digital scale (Seca 874 dr, Hamburg, Germany) and a flexible metric measuring tape (0.1 cm resolution). Parameters measured were height, weight, and BMI; bilateral arm length (acromion to olecranon); chest width (anterior axillary line to contralateral line); thoracic circumference at the xiphoid level; nipple and areolar diameters; sternal notch-to-nipple distances; and arm midpoint-to-nipple and nipple-to-infrapectoral fold distances (Figure 1).

For quality control, two trained investigators measured each parameter independently; discrepancies > 0.5 cm were rechecked jointly.

Statistical analysis

Normality was assessed with the Kolmogorov-Smirnov test. Because most variables were non-Gaussian, data are presented as medians and interquartile ranges. Group comparisons were performed using the Kruskal-Wallis test (for > 2 BMI groups). A two-tailed α = 0.05 was considered statistically significant. Analyses were performed using IBM SPSS Statistics for Windows, Version 28 (Released 2021; IBM Corp., Armonk, New York).

## Results

A total of 100 cisgender male patients were assessed between January and June 2024. All measurements and demographic data were collected by the same physician to ensure consistency. The average age was 29.6 ± 4.0 years, the average height 1.76 ± 0.05 meters, and the mean BMI 25 ± 2.8 kg/m² (Table 1).

**Table 1:** Sociodemographic data of participants

Variable	Category	Frequency (n)	Percentage (%)
Age	18–35 years	88	88%
	>35 years	12	12%
BMI	<25 kg/m²	57	57%
	25–30 kg/m²	39	39%
	>30 kg/m²	4	4%

NAC dimensions

The mean areolar height was 2.49 ± 0.55 cm (right) and 2.51 ± 0.58 cm (left), while the average areolar width measured 2.53 ± 0.52 cm (right) and 2.47 ± 0.48 cm (left). Nipple height was 0.50 ± 0.11 cm (right) and 0.50 ± 0.10 cm (left), with corresponding widths of 0.50 ± 0.13 cm and 0.52 ± 0.14 cm, respectively.

Thoracic metrics

The mean inter-nipple distance (IND) was 22.8 ± 2.3 cm. The distance from the sternal notch to the nipple was 20.8 ± 1.79 cm on the right and 21.07 ± 1.80 cm on the left. Chest width, measured between the anterior axillary lines, averaged 38.09 ± 4.6 cm, and thoracic circumference at the xiphoid process level was 93 ± 8.2 cm.

Arm length and nipple alignment

The average arm length was 38.46 ± 1.7 cm (right) and 38.3 ± 1.7 cm (left). The vertical deviation between the arm's mid-third and the ipsilateral nipple was 4 ± 13 mm on the right and 18 ± 11 mm on the left. The areola-infrapectoral fold distance was 3.3 ± 0.9 cm (right) and 3.3 ± 0.87 cm (left).

Proportional relationships

The IND-to-chest width ratio was 60 ± 4%, and the thoracic circumference was 25 ± 1% (Table 2).

**Table 2:** Anthropometric measurements of the male nipple-areola complex and chest

Parameter	Mean ± SD
Right areola height (cm)	2.40 ± 0.55
Left areola height (cm)	2.50 ± 0.58
Right areola width (cm)	2.50 ± 0.52
Left areola width (cm)	2.47 ± 0.48
Right nipple height (cm)	0.50 ± 0.11
Left nipple height (cm)	0.53 ± 0.10
Right nipple width (cm)	0.50 ± 0.13
Left nipple width (cm)	0.52 ± 0.14
Inter-nipple distance (cm)	22.89 ± 2.39
Sternal notch to right nipple distance (cm)	20.80 ± 1.70
Sternal notch to left nipple distance (cm)	21.07 ± 1.80
Difference between mid-arm point and right nipple (cm)	0.40 ± 1.30
Difference between mid-arm point and left nipple (cm)	0.18 ± 1.10
Chest width (anterior axillary line to line, cm)	38.09 ± 4.60
Chest circumference (cm)	93.09 ± 8.20
Ratio: inter-nipple distance/chest width	0.25 ± 0.01
Ratio: inter-nipple distance/chest circumference	0.60 ± 0.04

Statistical inference

The Kolmogorov-Smirnov test indicated a non-normal distribution for all measured variables. Consequently, non-parametric methods were used. The Mann-Whitney U test compared measurements across age groups (Table 3), while the Kruskal-Wallis test evaluated intergroup variation based on BMI (Table 4).

**Table 3:** Mann–Whitney U test between age groups (18–35 years and over 35 years for all variables) The Mann–Whitney U test was used to compare anatomical measurements between groups. A p-value < 0.05 was considered statistically significant. U = Mann–Whitney U statistic; Z = standard score; NAC = nipple-areola complex.

Variable	U-value	Z-score	p-value
Right nipple height	494	-0.116	0.908
Right nipple width	480	-0.272	0.786
Left nipple height	376	-1.507	0.132
Left nipple width	452	-0.59	0.555
Inter-nipple distance	462	-0.469	0.639
Sternal notch to the right NAC	404	-1.119	0.263
Sternal notch to the left NAC	446	-0.65	0.516
Chest circumference	436	-0.756	0.449
Distance between lateral anterior axillary lines (LAA)	452	-0.579	0.562
Right arm length	466	-0.48	0.631
Δ Right arm midpoint–nipple	208	-3.295	0.1
Left arm length	484	-0.243	0.808
Δ Left arm midpoint–nipple	408	-1.089	0.276
Right NAC to the inferior pectoral sulcus	384	-1.342	0.18
Left NAC to inferior pectoral sulcus	420	-0.947	0.344
Ratio: nipple distance/chest circumference	336	-1.865	0.062
Ratio: nipple distance/LAA distance	424	-0.888	0.375

**Table 4:** Kruskal–Wallis test for BMI *Comparisons between BMI groups were performed using the Kruskal–Wallis test for non-parametric data. Data are presented as means. A p-value < 0.05 was considered statistically significant. BMI = body mass index; NAC = nipple-areola complex; LAA = lateral anterior axillary lines.

Variable	< 25 (Mean)	25–30 (Mean)	> 30 (Mean)	Kruskal–Wallis H	p-value*
Right nipple height	0.46	0.56	0.60	20.79	< 0.01
Right nipple width	0.45	0.58	0.60	26.48	< 0.01
Left nipple height	0.51	0.57	0.60	13.28	< 0.01
Left nipple width	0.47	0.60	0.60	20.42	< 0.01
Inter-nipple distance	21.77	24.40	24.50	33.69	< 0.01
Sternal notch to the right NAC	19.89	21.88	24.00	40.54	< 0.01
Sternal notch to the left NAC	20.00	22.41	23.00	57.70	< 0.01
Chest circumference	88.54	98.27	109.00	42.86	< 0.01
Distance between lateral anterior axillary lines (LAA)	36.23	40.38	44.00	33.64	< 0.01
Right arm length	38.09	38.92	39.00	6.17	0.05
Δ Right arm midpoint–nipple	0.37	0.30	2.50	6.50	0.04
Left arm length	38.20	38.54	39.00	2.02	0.37
Δ Left arm midpoint–nipple	-0.10	0.26	3.50	11.06	< 0.01
Right NAC to the inferior pectoral sulcus	3.00	3.95	2.50	26.98	< 0.01
Left NAC to inferior pectoral sulcus	2.96	3.94	2.50	28.54	< 0.01
Ratio: nipple distance/chest circumference	0.25	0.25	0.22	7.62	0.02
Ratio: nipple distance/LAA distance	0.60	0.61	0.56	5.10	0.08

## Discussion

While the primary function of the nipple and areola-lactation is relevant only in women, aesthetic considerations of the male NAC have become increasingly important in procedures such as gynecomastia correction and female-to-male gender-affirming surgery. Unlike women, men often expose their chest during physical activity, making postoperative satisfaction with NAC appearance essential [22,23].

**Figure 2:**
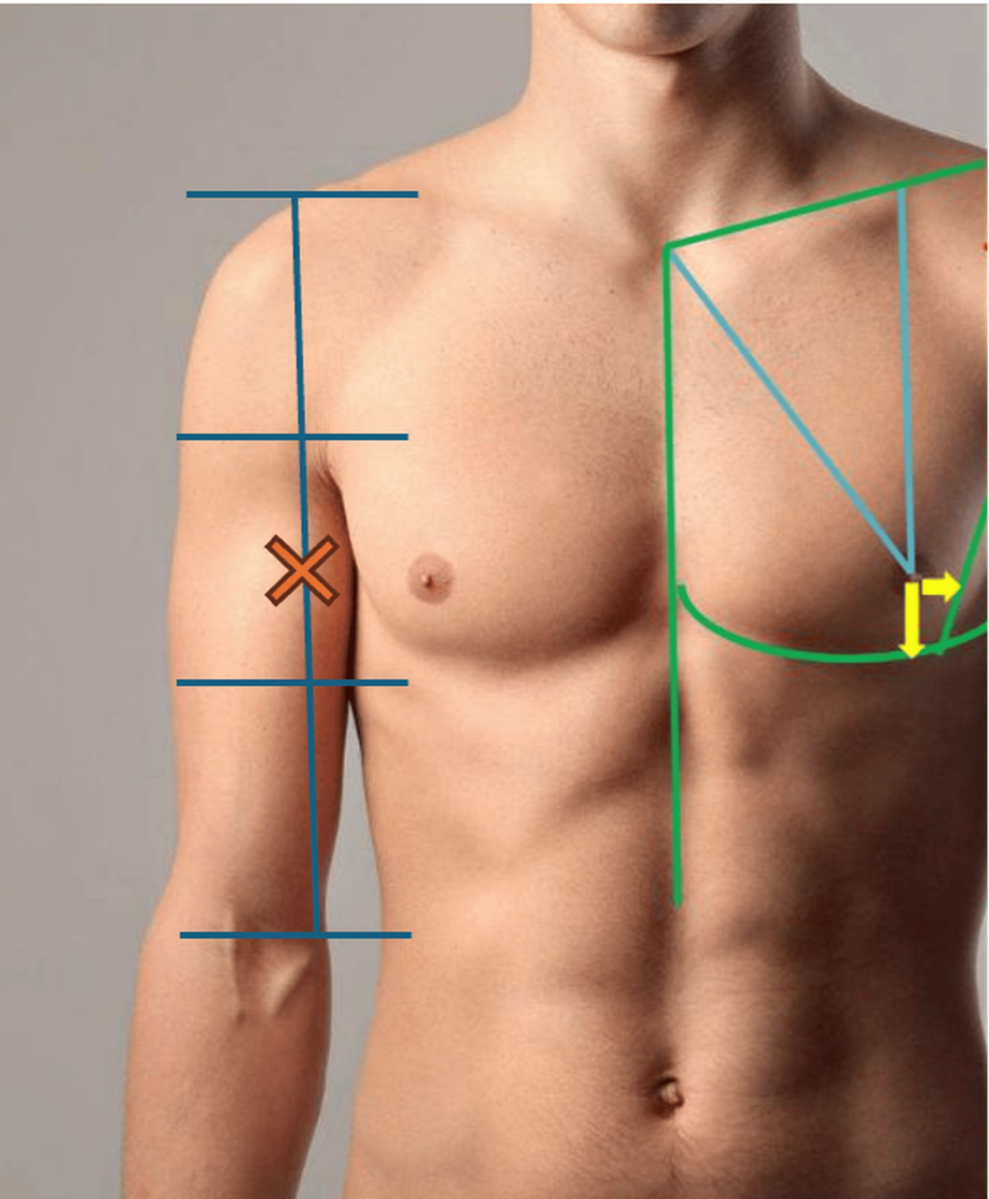
Anatomical landmarks used to evaluate the position of the nipple-areola complex (NAC) in relation to the arm and thorax. Depicted are the division of the upper arm into three equal horizontal thirds (blue lines), the vertical axis of the arm, and the ideal projected location of the NAC (marked with an "X") aligned with the junction between the middle third of the arm and the subpectoral fold. Reference lines are also shown for measurements from the sternal notch (blue and green lines) and the infrapectoral fold (curved green line). These anatomical relationships provide proportional guidelines for aesthetically positioning the NAC, with clinical applications in male chest contouring procedures such as masculinizing top surgery and gynecomastia correction. Original image by the authors.

Anatomical characteristics of the male NAC vary across populations and are influenced by genetics, body composition, and ethnicity [21]. However, data specific to the Mexican male population are scarce. Our study provides useful reference values for NAC size and location that may enhance intraoperative decision-making. Based on our findings, we propose a simple method to maintain proportionality between thoracic structures by using arm length as a reference (see Figure 2).

In our study, we observed that the vertical nipple position follows a consistent ratio relative to arm length, and the IND consistently measured approximately 60% of chest width. Importantly, these proportions remained consistent across BMI subgroups, supporting their potential clinical utility.

As BMI increased, nipples tended to shift laterally and inferiorly, consistent with the findings of Kasai et al. [5]. While anatomical landmarks, such as the sternal notch or anterior axillary line, may guide initial placement, further adjustments based on the patient's habitus are often necessary. In overweight patients, modest lateral repositioning may improve symmetry, though excessive movement can result in an unnatural appearance.

Patients with higher BMI also had larger areolas and nipples. This reinforces the need for individualized NAC design (larger in overweight patients, smaller in leaner ones) to maintain aesthetic balance. Table 5 summarizes the recommended distances from the sternal notch to the nipple and the IND across different BMI categories in our sample.

**Table 5:** Suggested sternal notch–nipple and inter-nipple distances according to BMI According to the results, and to facilitate the positioning of the nipple-areola complex in our population, we suggest adjusting it based on BMI and the surgeon's clinical judgment, following the table below.

BMI (kg/m²)	Suggested sternal notch–nipple distance (cm)	Suggested inter-nipple distance (cm)
< 25	20	22
25–30	22	24
> 30	23.5	25

According to these results, we suggest adjusting NAC positioning based on BMI and the surgeon's clinical judgment, as outlined above. Unlike formulas that rely on chest circumference, which can be difficult to assess intraoperatively, especially during glandular excision, our approach uses chest width and arm length, which are easily measurable in the operating room. Ratios such as IND-to-chest width and nipple height-to-arm length remained stable, offering practical intraoperative value.

Various authors have proposed formulaic approaches to nipple positioning. Shulman et al. [18] based their method on height and chest circumference; Beer et al. [6] used thoracic circumference and sternal length; Atiyeh et al. [17] incorporated the sternal notch, umbilicus, and axilla; McGregor [19,20] relied on multiple anatomical landmarks; and Tanini et al. [13] emphasized fixed relationships with the pectoralis major. However, Monstrey et al. [11] cautioned that absolute measurements may be misleading, and Murphy et al. [15] stressed the importance of proportionality over fixed values.

In our experience, aesthetic judgment and intraoperative flexibility yield better outcomes than strict adherence to formulas. Although early techniques often relied on visual estimation, modern practice still benefits from surgical intuition [14].

This study is limited by its relatively small, single-center sample, which is predominantly composed of individuals aged 20 to 35 years, with few participants presenting a BMI greater than 30. Future studies with larger, more diverse populations are necessary to validate these findings and refine anthropometric guidelines for NAC positioning.

## Conclusions

This study provides the first set of anthropometric reference values for the male NAC in a Mexican population. These data may assist surgeons in achieving more accurate, symmetrical, and culturally relevant results in both aesthetic and gender-affirming chest procedures. A notable strength of this study lies in the use of standardized measurement techniques, consistently applied by the same evaluators to enhance reliability. Nevertheless, the study has certain limitations. It was conducted at a single center with a relatively modest sample size, and individuals with a BMI >30 were underrepresented. Caution is therefore warranted when generalizing these findings to broader or more diverse populations.

By systematically documenting NAC size and position in relation to anatomical landmarks, this study offers practical and reproducible reference values for use in procedures such as gender-affirming mastectomy and gynecomastia surgery. The stability of key ratios, such as the IND-to-chest width ratio and nipple position relative to arm length, supports their intraoperative utility across varying body types. These findings may contribute to improved surgical planning, enhanced aesthetic outcomes, and increased patient satisfaction in male chest reconstruction.
